# Effect of previous stroke on quality of inpatient care and long-term mortality risk of non-ST-segment myocardial infarction

**DOI:** 10.1093/ehjopen/oeaf143

**Published:** 2025-10-24

**Authors:** Andrew Cole, Nicholas Weight, Mustafa Al-Jarshawi, Muhammad Rashid, Mamas A Mamas

**Affiliations:** Keele Cardiovascular Research Group, Centre for Prognosis Research, Institute for Primary Care and Health Sciences, Keele University, Staffordshire ST5 5BG, UK; Keele Cardiovascular Research Group, Centre for Prognosis Research, Institute for Primary Care and Health Sciences, Keele University, Staffordshire ST5 5BG, UK; Keele Cardiovascular Research Group, Centre for Prognosis Research, Institute for Primary Care and Health Sciences, Keele University, Staffordshire ST5 5BG, UK; Keele Cardiovascular Research Group, Centre for Prognosis Research, Institute for Primary Care and Health Sciences, Keele University, Staffordshire ST5 5BG, UK; Department of Cardiovascular Sciences, University of Leicester, Leicester LE1 7RH, UK; National Institute for Health Research (NIHR) Leicester Cardiovascular Biomedical Research Unit, Glenfield Hospital, Leicester LE3 9QP, UK; Keele Cardiovascular Research Group, Centre for Prognosis Research, Institute for Primary Care and Health Sciences, Keele University, Staffordshire ST5 5BG, UK; National Institute for Health and Care Research (NIHR) Birmingham Biomedical Research Centre, Institute of Translational Medicine, Birmingham B15 2TH, UK

**Keywords:** Stroke, NSTEMI, Quality of Care, Epidemiology, Outcomes

## Abstract

**Aims:**

Individuals with a previous stroke face an increased risk of Non-ST-segment myocardial infarction (NSTEMI) and may have a higher associated mortality. However, the impact of inpatient care quality during the NSTEMI admission on long-term outcomes remains unclear. To assess whether there were disparities in care and NSTEMI clinical outcomes between individuals with and without a previous stroke.

**Methods and results:**

We analysed 425 274 adults hospitalized between January 2005 and March 2019, with NSTEMI from the UK Myocardial Ischaemia National Audit Project (MINAP) registry, linked with Office for National Statistics mortality reporting. We examined long-term outcomes by previous stroke status and inpatient care quality for patients that survived to discharge using the opportunity-based quality-indicator score (OBQI) score, categorized as ‘poor’, ‘fair’, ‘good’ or ‘excellent’. Individuals with previous stroke were older (median age 79 vs. 72 years) and underwent revascularization by PCI (22% vs. 37%) less frequently than those without a previous stroke. The adjusted mortality risk for those with a previous stroke was higher at 30 days (aHR 1.14, 95% CI 1.10, 1.18), 1 year (aHR 1.20, 95% CI 1.17, 1.22) and 10 years (aHR 1.27, 95% CI 1.26 1.29) with higher quality inpatient care associated with lower mortality rates compared with poor care (good: HR 0.86, 95% CI 0.80, 0.92; excellent: HR 0.76, 95% CI 0.71, 0.81).

**Conclusion:**

Individuals with a previous stroke, experience disparities during inpatient care following NSTEMI and have a higher risk of long-term mortality. Higher quality inpatient care may lead to better long-term survival.

## Graphical Abstract

**Graphical Abstract:**
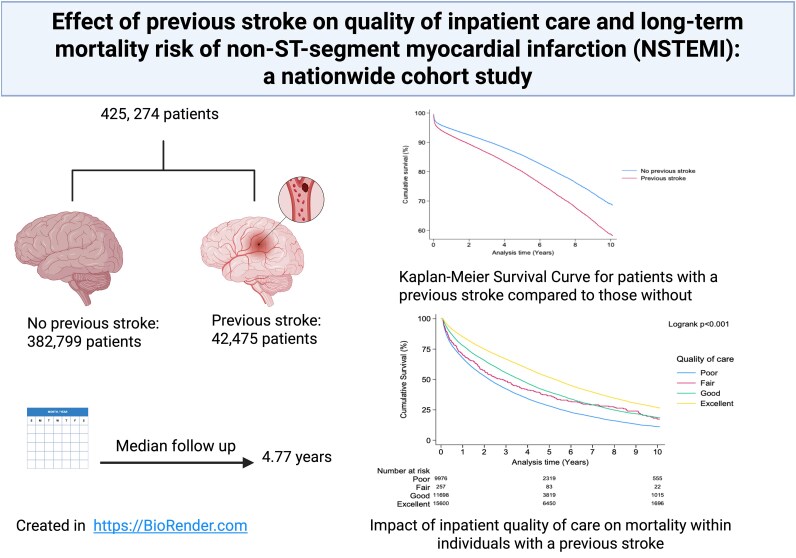

## Introduction

The current global prevalence of stroke is estimated to be around 100 million^1^ with the number of affected people projected to increase by up to 60% by 2035.^2^ Advances within hyperacute stroke care, including rapid assessment pathways, thrombolytic therapy and mechanical thrombectomy^3^ have improved the overall mortality^4^ associated with stroke, however acute myocardial infarction (AMI) remains a leading cause of mortality within this patient population.^5^ Within AMI, proportionally non-ST-segment myocardial infarction (NSTEMI) is more prevalent than ST-segment myocardial infarction^6^ and therefore it is anticipated that there will be increasing amounts of patients presenting with NSTEMI with a previous history of stroke.

Individuals with a previous stroke are at a greater risk of NSTEMI, which is mostly attributable to their shared cardiovascular risk profile.^7^ Additionally, NSTEMI mortality outcomes have been shown to be worse within individuals with a previous stroke. A small cohort study of 2548 patients suggested that this increased mortality risk exists up to 10 years of follow-up.^8^ Nevertheless, there have been no large, contemporary cohort studies assessing ACS treatments of patients with prior stroke, the quality of care received or their clinical outcomes. There is evidence that individuals with a previous stroke receive suboptimal, guideline directed, therapy during their inpatient stay.^9^ However, studies that assessed these disparities did so prior to the modern angioplasty era, which therefore limit their applicability to current patient populations.

We aimed to study whether there were disparities in care and clinical outcomes within a contemporary, large national AMI cohort. To address this, we used data from the Myocardial Ischaemia National Audit Project (MINAP) registry, linked to Office for National Statistics (ONS) mortality data, to compare the in-hospital quality of care that individuals receive according to previous stroke status, and to assess whether this influences their long-term mortality risk.

## Methods

### Study design

We used the MINAP registry, a prospective national registry of patients admitted to UK hospitals with an acute coronary syndrome (ACS).^10^ The MINAP registry is one the world’s largest AMI registries and consists of 130 variables, including baseline demographics, clinical characteristics, comorbidities, management strategies, pharmacotherapy, in-hospital clinical outcomes, and discharge diagnosis.^11^ Data are submitted by hospital clinical staff, and ∼90 000 pseudonymised records annually are uploaded to the National Institute for Cardiovascular Outcomes Research (NICOR). In-hospital mortality is recorded in the MINAP registry, but for out-of-hospital outcomes we used linked Office of National Statistics data, which is the UK’s independent provider of official statistics, regularly collecting data on every death registered in the UK, coding deaths according to the international classification of diseases (ICD-10) and cause of death from the medical certificate of cause of death.

### Study population

**Figure 1:**
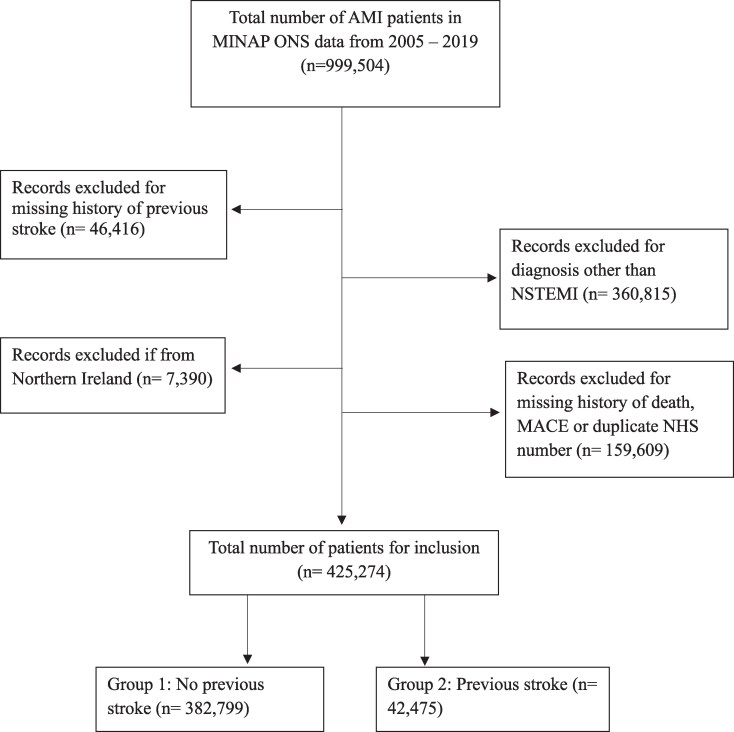
STROBE diagram detailing exclusion criteria.

We included patients admitted with a diagnosis of NSTEMI in any of the 230 participating hospitals in England and Wales between 1 January 2005 and 30 March 2019. The discharge diagnosis of NSTEMI was determined by local clinicians according to presenting history, clinical examination, and the results of in-patient investigations in keeping with the consensus document of the Joint European Society of Cardiology (ESC) and American College of Cardiology (ACC) Committee.^12^ Previous history of stroke, defined as either ischaemic or haemorrhagic stroke, was identified by clinicians during admission and included within MINAP data collection. Participants gender and ethnicity was self-reported during their inpatient admission. Patients were excluded if they had missing data for key variables of interest; diagnosis of previous stroke, in-hospital mortality, major adverse cardiovascular events (composite of inpatient death and reinfarction) and cardiac mortality risk. Patients with inconsistent recording of mortality dates were excluded. Patients’ index admission with NSTEMI was used for analysis purposes, with duplicate patient admissions excluded according to National Health Service (NHS) number. Records with a missing NHS number were also excluded. Mortality follow-up data was available from the ONS up to July 2021 via a single download (*Figure 1*).

### Outcomes

The primary outcome was assessment of inpatient quality of care and assessment of this on long-term mortality outcomes. To assess quality of NSTEMI case we utilized the opportunity-based quality indicator (OBQI) score, which comprises of inpatient prescription of aspirin, P2Y_12_ inhibitors, statins, β-blockers, angiotensin-converting enzyme inhibitors (ACEi) or angiotensin receptor blockers (ARB) and referral for cardiac rehabilitation.^13^ These represent elements of the European Society of Cardiology (ESC) quality metrics and form part of the 2023 ESC AMI guidelines.^14^ We classified the OBQI scores into four categories: ‘excellent’ refers to an OBQI (score of *≥*90 and ≤100); ‘good’ (*≥*80 and < 90); ‘fair’ (*≥*70 and <80), and ‘poor’ (<70).^15^ All-cause mortality was then assessed at 30-day, 1-year, 5-year and 10-year mortality (where available). All-cause mortality was calculated from the date of admission with AMI, as recorded in the MINAP registry, and the date of registration of death, as recorded by the ONS.

We also assessed the 2020 ESC Association for Acute Cardiovascular Care (ACVC) quality indicators for NSTEMI,^16^ including whether participants underwent invasive coronary angiography (ICA) within 72 h, received dual anti-platelet therapy at discharge, received low molecular weight heparin (LMWH) or fondaparinux and whether left ventricular function was assessed during admission.

### Statistical analysis

Demographics, clinical characteristics, and crude risks for adverse outcomes by presence of previous stroke were compared using Pearson’s chi-square test for categorical variables. Continuous variables were compared using Student's *t*-test, if normally distributed, and using the Wilcoxon Rank Sum test or Kruskal-Wallis test if not. The normality of distribution was assessed using the Shapiro-Wilk test. Continuous variables are presented as medians and interquartile ranges (IQR) and categorical variables by proportions. Multiple imputations with chained equations (MICE) were used to impute values for variables with missing data. MICE is the best practice when dealing with missing data and can provide unbiased estimates even when levels of missing data are significant and some protection when the pattern of ‘missingness’ is not random. Kaplan-Meier curves were plotted and Cox regression models were fitted (on ten imputed datasets), adjusted for; age, gender, year, ethnicity, comorbidities (diabetes mellitus, peripheral vascular disease, asthma or chronic obstructive pulmonary disease, chronic kidney disease, hypertension, smoking status and history of previous myocardial infarction), medication strategy (aspirin, P2Y12 inhibitor, LMWH, statin, warfarin, ACE inhibitor and β− blockers), clinical characteristics [admission ward, left ventricular ejection fraction, cardiac arrest, ICA or revascularization by percutaneous coronary intervention (PCI) and coronary artery bypass graft surgery (CABG)], to calculate hazard ratios (HR) for mortality risks associated with previous stroke. For temporal trend of 1-year mortality, study years were organized biennially, into 2005–2006, 2007–2008, 2009–2010, 2011–2012, 2013–2014, 2015–2016, and 2017–2018 for ease of presentation.

We modelled potential lives saved in a dataset extracted from our imputed dataset, running a logistic regression model, adjusted for the same variables as the previously described cox-model, then by applying the margins function on Stata 18.0 to calculate the adjusted 1-year mortality for each category of OBQI score. We then applied the difference in adjusted mortality between different OBQI score categories to the population at risk, to model the potential mortality benefit at 1 year of receiving ‘excellent’ care according OBQI score.

Stata 18.0 was used for all analyses and an alpha level of 0.05 was used throughout.

## Results

After applying the relevant exclusion criteria, between January 2005 and March 2019, of patients admitted to England and Wales hospitals with an AMI, there were 425 274 individuals with a diagnosis of NSTEMI (*Figure 1*). Of these, 42 475 (10%) individuals had a diagnosis of previous stroke. The median duration of follow-up for patients included in the study was 4.77 years (IQR: 2.1–8.6 years).

### Demographic comparison between NSTEMI participants with and without previous stroke

Individuals with a previous stroke were older (median age: 79.3, IQR 71.4–85.5 years vs. 72.3, IQR 61.1–81.6 years) and were more likely to be female (41% vs. 36%, *P* < 0.001) (*Table 1*).

**Table 1:** Demographic comparison between patients presenting with NSTEMI with and without previous stroke

Variable	No previous Stroke (*n* = 382 799)	Previous Stroke (*n* = 42 475)	*P* value
Age (years)	72.3 (61.1–81.6)	79.3 (71.4–85.5)	<0.001
Female	138 259/382 799 (36)	17 438/42 475 (41)	<0.001
BMI (kg/m^2^)	27.1 (24.0–30.8)	26.3 (23.1–30.1)	<0.001
Ethnicity			<0.001
White	182 866/198 576 (92)	19 943/21 492 (93)	
Asian	13 085/198 576 (7)	1200/21 492 (6)	
Black	2167/198 576 (1)	311/21 492 (1)	
Mixed	458/198 576 (0)	38/21 492 (0)	
Killip Class			<0.001
Basal crepitations	26 065/179 975 (14)	4457/19 084 (23)	
Pulmonary oedema	9442/179 975 (5)	1748/19 084 (9)	
Cardiogenic shock	943/179 975 (1)	137/19 084 (1)	
GRACE score			<0.001
High risk (>140)	105 872/175 475 (60)	14 952/18 588 (80)	
Intermediate risk (109–140)	46 645/175 475 (27)	2895/18 588 (16)	
Low risk (<109)	22 958/175 475 (13)	741/18 588 (4)	
Previous smoker	133 684/363 286 (37)	16 444/38 982 (42)	<0.001
Current smoker	83 619/363 286 (23)	5889/38 982 (15)	<0.001
CCF	25 424/380 469 (7)	5276/41 631 (13)	<0.001
Hypercholesterolaemia	125 025/375 830 (33)	16 119/41 024 (39)	<0.001
Diabetes Mellitus	89 523/379 457 (24)	13 561/41 948 (32)	<0.001
Chronic kidney disease	26 501/380 683 (7)	5772/41 638 (14)	<0.001
History of angina	107 458/379 953 (28)	16 260/41 689 (39)	<0.001
Peripheral vascular disease	17 461/377 948 (5)	4226/41 389 (10)	<0.001
Hypertension	201 005/380 894 (53)	27 510/41 985 (66)	<0.001
Asthma/COPD	64 028/378 107 (17)	8408/41 580 (20)	<0.001
Previous AMI	88 252/380 795 (23)	14 382/41 910 (34)	<0.001
Previous PCI	39 047/377 651 (10)	4356/41 418 (11)	0.260
Previous CABG	28 019/378 330 (7)	4329/41 623 (10)	<0.001
Family history of CAD	92 070/309 496 (30)	6410/30 799 (21)	<0.001
Heart rate (bpm)	79 (67–93)	81 (69–97)	<0.001
Systolic BP (mmHg)	140 (122–159)	139 (120–159)	<0.001
LV function^a^			<0.001
Good	105 812/280 787 (38)	9175/30 965 (30)	
Moderate	47 935/280 787 (17)	5701/30 965 (18)	
Severe	18 664/280 787 (7)	2843/30 965 (9)	
Cardiac arrest	12 423/373 350 (3)	1848/41 449 (5)	<0.001
Admission under cardiologist	169 468/378 872 (45)	15 142/42 105 (36)	<0.001
Admission to cardiology ward^b^	195 280/380 818 (51)	17 659/42 267 (42)	<0.001

BMI, body mass index; Bpm, beats per minute; CABG, coronary artery bypass graft; CCF, congestive cardiac failure; COPD, chronic obstructive pulmonary disease; GRACE, global registry of acute coronary events; IQR, interquartile range; LV, left ventricular; MI, myocardial infarction.

Continuous variables are expressed as median (IQR) and categorical variables as proportions (%). Denominators represent the total number of participants with a data point collected; numerators represent the number of those participants for whom the variable of interest was present. Cardiac arrest is a composite of both in-hospital and out of hospital cardiac arrests.

^a^Good left ventricular function was defined as an ejection fraction (EF) ≥ 50%, moderate LV function as an EF 30–49% and severe LV function as an EF <30%.

^b^Admission to cardiology ward’ is a composite of admission to a coronary care unit or a general cardiology ward.

Chronic kidney disease is recorded in the MINAP registry as a serum creatinine level chronically elevated above 200 µmol/L.

Individuals with a previous stroke were more likely to present with common cardiovascular risk factors including, hypercholesterolaemia (39% vs. 33%, *P* < 0.001), chronic kidney disease (14% vs. 7%, *P* < 0.001), peripheral vascular disease (10% vs. 5%, *P* < 0.001), diabetes (32% vs. 24%, *P* < 0.001), hypertension (66% vs. 53%, *P* < 0.001) and a history of previous AMI (34% vs. 23%, *P* < 0.001). Individuals with a previous stroke were less likely to be admitted under a cardiologist (36% vs. 45%, *P* < 0.001) (*Table 1*).

### Management strategies and unadjusted clinical outcomes for NSTEMI participants with and without a previous stroke

Individuals with a previous stroke were less likely to receive revascularization by PCI (19% vs. 34%, *P* < 0.001) and were less likely to receive β-blockers (74% vs. 79%, *P* < 0.001). Their unadjusted MACE was higher than for individuals without a previous stroke (11% vs. 6%, *P* < 0.001) (*Table 2*).

**Table 2:** Management strategy and outcome comparison between patients presenting with NSTEMI with and without previous stroke

Variable	No previous Stroke (*n* = 382 799)	Previous Stroke (*n* = 42 475)	*P* value
LMWH	205 235/343 679 (60)	22 951/38 370 (60)	0.711
Fondaparinux	125 677/299 223 (42)	12 201/33 220 (37)	<0.001
Warfarin	19 329/341 170 (6)	4367/38 199 (11)	<0.001
Unfractionated heparin	45 573/339 720 (13)	3797/38 052 (10)	<0.001
Glycoprotein 2b/3a inhibitor	13 961/344 652 (4)	885/38 548 (2)	<0.001
Intravenous nitrate	48 155/341 145 (14)	5298/38 127 (14)	0.242
Furosemide	17 515/340 806 (5)	2459/38 133 (6)	<0.001
MRAs	16356/253 052 (6)	2461/27 832 (9)	<0.001
Aspirin	367 241/380/540 (97)	39 626/42 170 (94)	<0.001
P2Y_12_ inhibitors	322 859/374 773 (86)	34 377/41 413 (83)	<0.001
Statins	313 315/379 299 (83)	35 730/42 078 (85)	<0.001
ACE inhibitors/ARBs	277 425/378 421 (73)	30 111/41 975 (72)	<0.001
β-blockers	300 158/379 262 (79)	31 071/42 057 (74)	<0.001
ICA	236 639/370 243 (64)	16 808/40 868 (41)	<0.001
PCI	113 887/304 469 (37)	7139/32 466 (22)	<0.001
CABG surgery	11 174/304 469 (4)	769/32 466 (2)	<0.001
Revascularization (CABG surgery/PCI)	125 679/375 050 (34)	7973/41 703 (19)	<0.001
In-hospital mortality	20 719/382 799 (5)	4235/42 475 (10)	<0.001
30-day mortality	24 698/382 799 (6)	4921/42 475 (12)	<0.001
1 year mortality	63 931/382 799 (17)	13 132/42 475 (31)	<0.001
5-year mortality	115 815/308 266 (38)	22 464/35 159 (64)	<0.001
10-year mortality	95 663/169 039 (57)	16 562/19 860 (83)	<0.001
Inpatient cardiac mortality	15 876/382 799 (4)	5155/42 475 (7)	<0.001
Reinfarction	3632/354 932 (1)	528/39 451 (1)	<0.001
Major bleeding	5605/372 261 (2)	852/41 377 (2)	<0.001
MACE^a^	23 566/382 799 (6)	4623/42 475 (11)	<0.001
Circulatory cause of death	74 280/183 503 (40)	12 791/31 576 (41)	0.921

Data are presented as proportions (%). Denominators represent the total number of participants with a data point collected; numerators represent the number of those participants for whom the variable of interest was present.

^a^MACE was defined as a composite endpoint of in-hospital death and reinfarction.

ACE, angiotensin-converting-enzyme; ARB, angiotensin receptor blockers; CABG, coronary artery bypass graft; ICA, invasive coronary angiography; LMWH, low molecular weight heparin; MRA, mineralocorticoid receptor antagonist; PCI, percutaneous coronary intervention.

**Figure 2:**
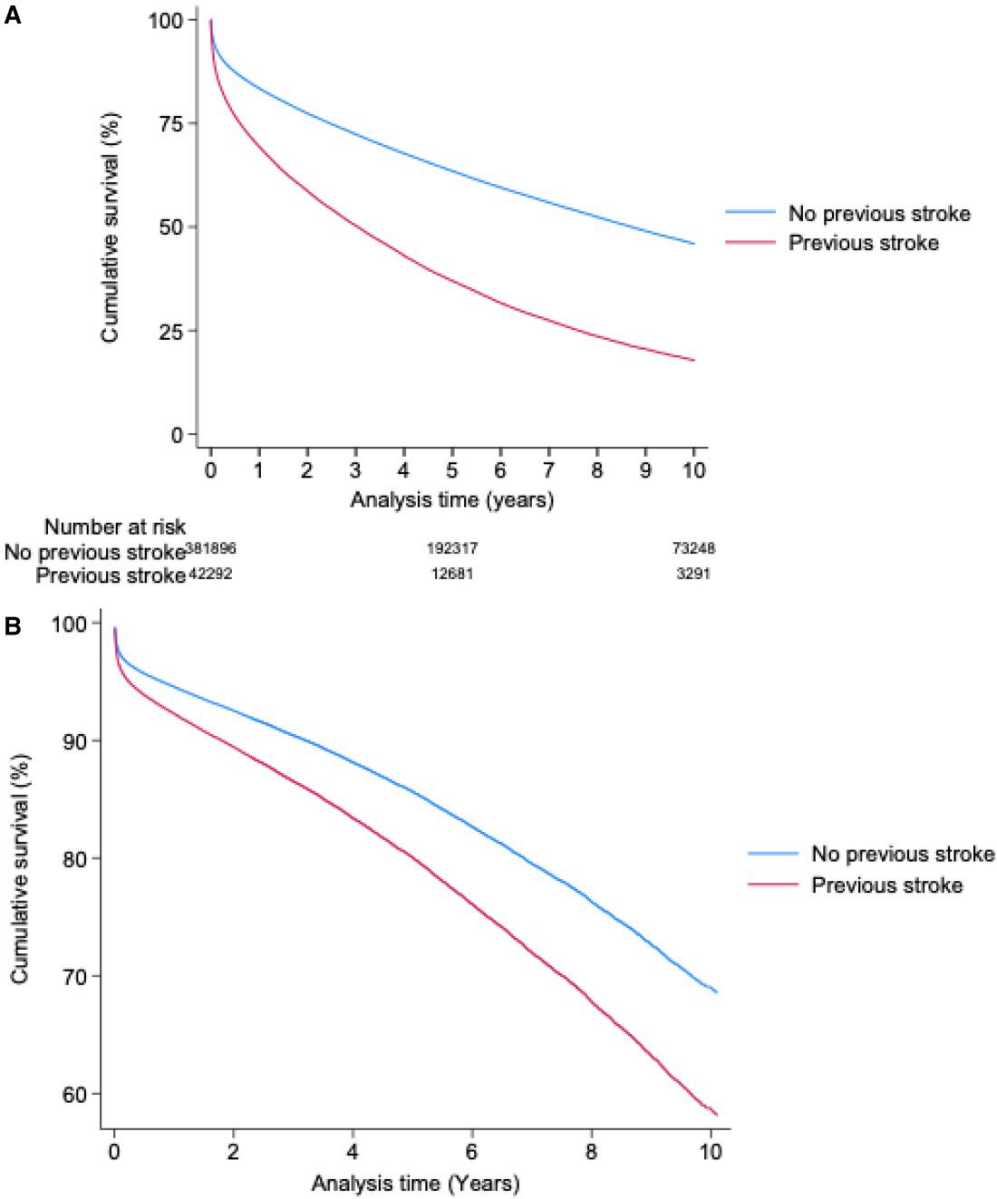
Kaplan-meier survival curve for patients with a previous stroke compared with those without. (*A*) Unadjusted Kaplan-Meier Survival Curves. (*B*) Adjusted Kaplan-Meier Survival Curves adjusted for: age, gender, year, ethnicity, heart rate, blood pressure, co-morbid conditions (diabetes mellitus, hypertension, history of asthma or COPD, history of PVD, history of CKD, previous AMI and smoking status), pharmacotherapy (prescription of LMWH, warfarin, aspirin, P2Y12 inhibitor, statin, ACE inhibitors and beta blockers), Left ventricular ejection fraction, cardiac arrest and procedures including coronary angiography during admission and revascularization (by PCI or CABG during admission). MACE is defined as composite endpoint of in-hospital death and reinfarction.

When compared with individuals without a previous stroke, unadjusted mortality over 30 days (12% vs. 6%, *P* < 0.001), 1 year (31% vs. 17%, *P* < 0.001), 5 years (64% vs. 38%, *P* < 0.001) and 10 years (83% vs. 57%, *P* < 0.001) were all higher in individuals with a previous stroke (*Table 2*) (*Figure 2*).

### Quality indicators for NSTEMI participants with and without a previous stroke

Individuals with a previous stroke were less likely to receive a coronary angiogram within 72 h of admission (53% vs. 64%, *P* < 0.001) and were less likely to receive DAPT on discharge (79% vs. 84%, *P* < 0.001). For individuals with moderate to severe left ventricular systolic dysfunction, they were less likely to receive β-blockers (81% vs. 83%, *P* < 0.001) (*Table 3*). They were less likely to be referred for cardiac rehabilitation (66% vs. 79%, *P* < 0.001) and overall quality of care, as assessed by the OBQI score, was lower (79.9 vs. 83.2, *P* < 0.001). (*Table 3*).

**Table 3:** Quality indicators for patients presenting with NSTEMI with and without previous stroke (ESC ACVC and OBQI)

Variable	No previous Stroke (*n* = 382 799)	Previous Stroke (*n* = 42 475)	*P* value
Coronary Angiography received within 72 h (%)	30 895/48 519 (64)	1833/3453 (53)	<0.001
LV Function recorded in notes (%)	201 075/304 011 (66)	21 303/33 543 (64)	<0.001
Fondaparinux or LMWH received (%)	76 542/347 980 (22)	8174/38 796 (21)	<0.001
DAPT received on discharge (%)	315 125/374 351 (84)	32 863/41 382 (79)	<0.001
ACEi or ARB on discharge for those with moderate and severe LVSD (%)	51 165/66 260 (77)	6525/8516 (77)	0.216
β−Blocker on discharge for those for those with moderate and severe LVSD (%)	55 234/66 389 (83)	6879/8528 (81)	<0.001
Composite All/None score^a,b^ (%)	266 750/382 799 (69)	28 648/42 475 (67)	<0.001
Composite All/None score for those with moderate and severe LVSD (%)	49 140/66 599 (74)	6338/8544 (74)	0.433
Mean OBQI score^c^	83.2	79.9	<0.001
Cardiac rehabilitation (%)	278 637/352 324 (79)	25 852/38 948 (66)	<0.001

ACVC, Association for Acute Cardiovascular Care; ESC, European Society of Cardiology.

Data are expressed as proportions (%) unless indicated otherwise. Denominators represent the total number of participants with a data point collected; numerators represent the number of those participants for whom the variable of interest was present.

^a^MINAP does not record the specific type of statins, so ‘statin prescription’ was used as a surrogate for high intensity statin.

^b^Composite score of receipt of low dose aspirin, P2Y_12_ inhibition and statin.

^c^Opportunity based QI (The score consisted of 6 evidence-based processes of care: the prescription of aspirin, thienopyridine inhibitor, β-blocker, angiotensin converting enzyme inhibitor (ACEi), HMG CoA reductase enzyme inhibitor (statin) and enrolment onto a cardiac rehabilitation programme at the time of discharge). The OBQI reflects the number of care opportunities fulfilled at each hospital (numerator) divided by the number of opportunities to provide care (denominator). Excluded from both numerator and denominator were interventions that were contra-indicated, not applicable, not indicated in, or declined by, individual patients. Number of participants in each group and category: No previous stroke: Poor 100,125, Fair 1,837, Good 98,424, Excellent 182 081. Previous stroke: Poor 13,539, Fair 306, Good 12,542, Excellent 16 053.

### Long-term mortality analysis

The multivariable-adjusted risk of mortality was higher for individuals with a previous stroke at 30 days (aHR: 1.14, CI; 1.11–1.18, *P* < 0.001), 1 year, (aHR: 1.20, CI; 1.17–1.22, *P* < 0.001), 5 years, (aHR: 1.25, CI; 1.24–1.27, *P* < 0.001) and 10 years (aHR: 1.27, CI; 1.26–1.29, *P* < 0.001) (*Table 4*).

**Table 4:** Survival analysis for patients admitted with NSTEMI comparing outcomes in those with and without previous stroke

Outcomes	Adjusted hazard ratio for people with a previous stroke compared with those without (95% CIs)	*P*-value
30-day mortality	1.14 (1.11–1.18)	<0.001
1-year mortality	1.20 (1.17–1.22)	<0.001
5-year mortality	1.25 (1.24–1.27)	<0.001
10-year mortality	1.27 (1.26–1.29)	<0.001
Overall mortality	1.28 (1.26–1.29)	<0.001

Adjusted hazard ratios are presented with 95% CIs, adjusted for: age, gender, year, ethnicity, heart rate, blood pressure, co-morbid conditions (diabetes mellitus, hypertension, history of asthma or COPD, history of PVD, history of CKD, previous AMI and smoking status), pharmacotherapy (prescription of low molecular weight heparin (LMWH), warfarin, aspirin, P2Y12 inhibitor, statin, ACE inhibitors and beta blockers), Left ventricular ejection fraction, cardiac arrest and procedures including coronary angiography during admission and revascularization (by PCI or CABG during admission). MACE is defined as composite endpoint of in-hospital death and reinfarction.

**Figure 3:**
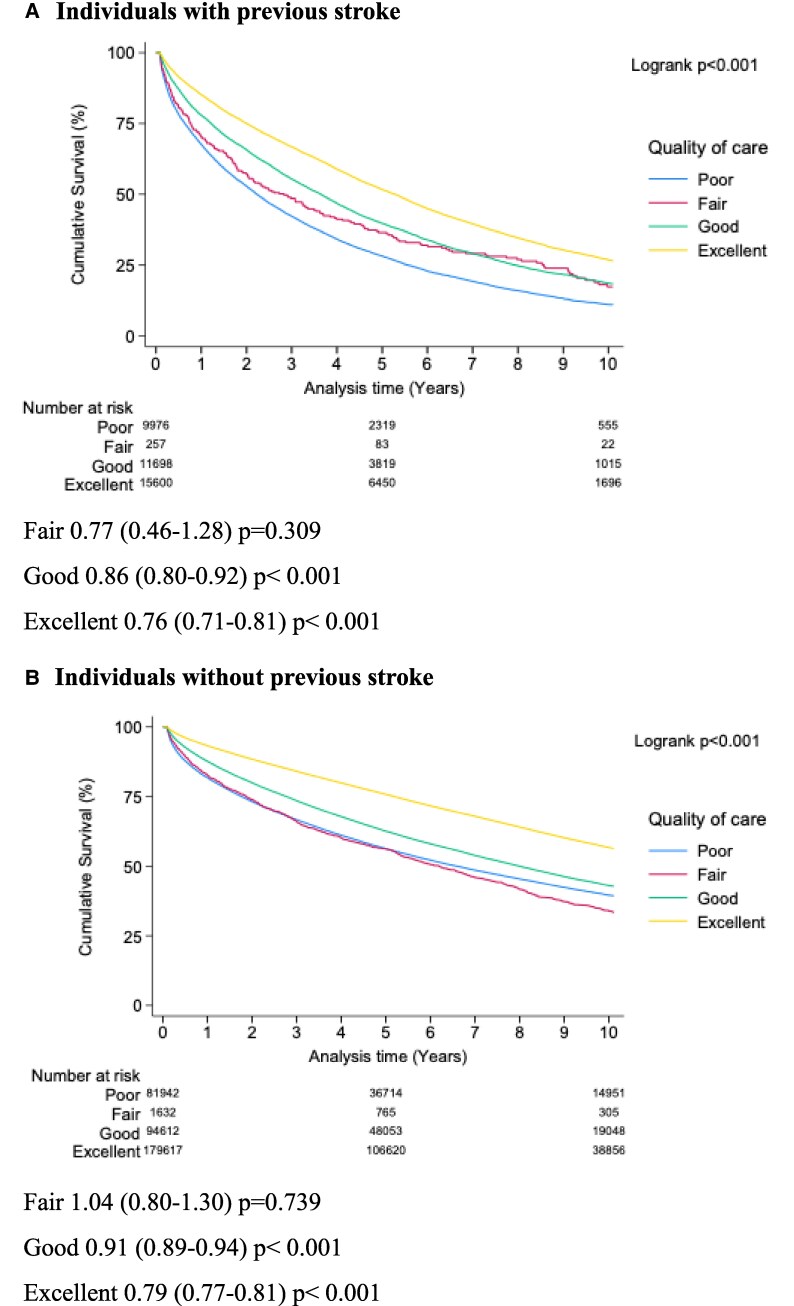
Impact of inpatient quality of care according to opportunity based quality indicator score (OBQI) on clinical outcomes of individuals with previous stroke. Results landmarked to exclude mortality within 30 days of admission to reflect aspects of OBQI score that are assessed on discharge.

Within individuals with a previous stroke, higher quality inpatient care, as derived from the OBQI score, led to a lower risk of long-term mortality, ‘good care’ (HR 0.86, CI 0.80–0.92, *P* < 0.001) and ‘excellent care’ (HR 0.76, CI 0.71–0.81, *P* < 0.001) (*Figure 3I*).

‘Excellent’ care was associated with better outcomes similarly in individuals with and without a previous stroke (previous stroke: HR 0.76, CI 0.71–0.81, No previous stroke: 0.79, CI 0.77–0.81) (*Figure 3I-II*).

Temporal trends in absolute risk for 1 year mortality improved similarly for individuals with and without a previous stroke (2005–2006: 35% vs. 20%; 2017–2018: 27% vs. 13%) (Supplementary material online, *Figure S1*).

*Table 5* outlines estimated potential lives saved if all participants received ‘excellent’ care. There is an anticipated survival benefit in improving care for participants receiving ‘poor’, ‘fair’ and ‘good’ care and proportionally this was greater in participants with a previous stroke. There was a potential for over 2702 lives to be saved during the whole study period if individuals with a previous stroke, received excellent care.

**Table 5:** Modelled lives saved if quality of care improved to excellent care according to previous stroke status

Stroke status	Quality of care	Total patients per group	Difference in mortality compared with excellent care group (%)	Modelled lives saved at one-year if all patients received excellent care
No previous Stroke	Poor	100 125	11	11 014
	Fair	1837	6	110
	Good	98 424	3	2953
Previous Stroke	Poor	13 539	16	2166
	Fair	306	11	34
	Good	12 542	4	502

Modelled lives saved calculated from assuming number of patients admitted NSTEMI with previous stroke increases in proportion if all patients receive excellent care.

## Discussion

Our analysis of over 420 000 patients presenting with NSTEMI reveals important disparities in care and outcomes between individuals with and without a previous stroke. Individuals with a previous stroke were older, had more cardiovascular co-morbidities and were less likely to undergo ICA during their admission. Individuals with a previous stroke received lower quality of care, were less likely to receive coronary angiography within 72 h and were less likely to receive DAPT or referral to cardiac rehabilitation on discharge. Overall, they had a higher risk of mortality from 30 days, to, up to, 10 years of follow-up. Importantly, this risk was lower within cohorts that received higher quality, guideline directed, care. We show that over 2700 lives could have been saved during our study period these individuals received excellent care.

Cardiovascular disease is the leading cause of death within individuals who have had a non-fatal stroke.^17^ The mechanisms and pathogenesis of stroke and NSTEMI share many similarities. With both involving the chronic development of atherosclerotic plaques and thus sharing similar co-morbid risk profiles. As expected, our study highlights this shared risk profile and shows that individuals with a previous stroke were more likely to present with typical cardiovascular risk factors such as CKD, hypercholesterolaemia, hypertension and diabetes. It is likely this shared risk profile not only contributes to the increased incidence of NSTEMI, but also partially explains the discrepancy, we show, in invasive management and longer-term mortality. For example, CKD has been shown to increase the risk of bleeding complications from invasive therapy,^18^ whilst hypertension and diabetes are independently associated with a higher long-term mortality risk after NSTEMI admission.^15,19^

Prior to this study, mortality risk within individuals with a previous stroke has been evaluated predominately within the context of heterogenous ACS cohorts. Takeuchi *et al*.^8^ showed within a single centre, 1999–2015 cohort of 2548 patients presenting with ACS, that patients with a previous stroke had a hazard ratio of 1.41 (95% CI 1.03, 1.93) of all-cause mortality. Whilst Buckley *et al*.^20^ showed within a US national 2002–2021 cohort of 365 383 patients presenting with stroke, that patients had a 49% higher chance of developing ACS and had a raised 5-year all-cause mortality with an odds ratio of 1.49 (95% CI, 1.44, 1.54). The only current NSTEMI specific cohort was assessed by Abtahian *et al*.^9^ This was a 2007 cohort of 25 514 patients’ which suggested that individuals with a previous stroke had worse in hospital mortality after presenting with NSTEMI. However, with an odds ratio of 1.11 (95% CI 0.92–1.38) this did not reach statistical significance. Importantly, therefore this study is the first large contemporary study which shows individuals with a previous stroke have a significantly worse mortality, which extends up to 10 years of follow-up.

The explanation of why these differences exist is likely multifactorial. Individuals with a previous stroke presented more commonly with adverse features of NSTEMI including pulmonary oedema, severe left ventricular impairment and cardiac arrest. These individual variables have been previously linked to increased mortality risk within NSTEMI.^21,22^ Furthermore, individuals with a previous stroke were less likely to be admitted to a specialist cardiology ward. Individuals are more likely to receive early guideline directed medical therapy when cared for in this setting.^13^ Patients within a cardiology ward are also more likely to receive specialist investigations such as echocardiograms and cardiac MRI. Availability of which would speed up the diagnosis and treatment of post-infarct complications, and prompt, more aggressive titration of heart failure therapy.^23,24^ Furthermore, cardiologists are more likely to utilize and understand the importance of referrals to cardiac rehabilitation,^25^ which may partially contribute to the lower rate of referral within individuals with a previous stroke. Whilst non-referral could be an active decision from the treating clinician based on neurological deficits, studies have shown the importance of cardiac rehabilitation in post-AMI care,^26^ and that programmes can be adapted for individuals with physical impairments.^27^

Our main finding in this study is that individuals with previous stroke receive poorer care during NSTEMI admission. One of the most striking disparities in our assessment of care, is the discrepancy in the proportion of patients that receive coronary angiography and invasive therapy. We suspect that this difference experienced by these patients may represent their associated risk factors. For example, the presence of impaired renal function may lead to concerns over contrast nephropathy and clinicians avoiding or delaying invasive therapy.^28^ Equally this could also represent a sequela of their more unstable presentations and requirement for stabilization prior to angiogram. Additionally, despite evidence that PCI does not worsen stroke outcomes if required for ACS,^29^ clinicians may feel exposure to high doses of anticoagulation too high risk in patients with a prior history of stroke, and that longer term treatment with potent anti-platelet agents following PCI may increase risk of further events.

We display within this study therefore that individuals with a previous stroke are at a higher risk of cardiovascular disease. They present acutely more unwell and with a greater number of cardiovascular co-morbidities. It is therefore surprising that this high-risk group of patients received the lowest frequency medical and invasive therapy. It is well documented in similar high-risk groups of patients that often the higher-risk individuals are less likely to receive guideline directed therapy. This discrepancy is likely driven over concerns of complications during procedures or from medications and has been termed a ‘risk-treatment paradox’.^30^ This leads to overly cautious management of patients, who possibly stand to benefit the most with treatment.^31^ Recognition and reversal of this paradox may be one way of improving clinical outcomes within individuals with a previous stroke.

### Strengths

To our knowledge, this is the largest, contemporary study of long-term NSTEMI survival in patients with a previous stroke and there are several strengths to this study. The MINAP registry collects national data, with many recorded variables from all individuals presenting to hospitals with NSTEMI in the UK. Linking our data to ONS mortality outcomes provides robust mortality data linkage. This allows for regional differences within the UK to be balanced out and therefore the results presented here are likely to be representative of other publicly funded healthcare models globally. Our post-discharge mortality data, with a median duration of follow-up of 4.77 years gives us a long follow-up period through which to assess mortality risks. Importantly our follow-up period has allowed us to make time weighted assessments of changes in mortality within a period that did not include the COVID-19 pandemic, wherein trends of mortality and morbidity were significantly altered.

### Limitations

This study has several limitations comparable to other large national databases. The MINAP database collects a multitude of variables but does lack data on frailty, treatment rationale, angiographic findings and a comprehensive list of comorbidities. MINAP also has strict definitions for comorbidities; for example, CKD is defined as creatinine >200 micromol/L, preventing subclassification by kidney disease severity. There is also no external validation of data inputs.

Data is collected at the point of NSTEMI diagnosis, without collecting date of previous stroke diagnosis therefore we are not able to assess for outcome differences depending on the duration between stroke and NSTEMI. MINAP also records stroke as a heterogenous term and does not differentiate between ischaemic or haemorrhagic stroke. Additionally, the MINAP registry does not collect data on participants functional baseline, and we are not able to comment on any residual neurological deficits that individuals may have.

Within our use of the OBQI score to assess quality of care, individuals were excluded if medications were recorded as clinically contraindicated or not applicable. Given the nature of data coding at the end of patients’ admissions, it is possible that medications were omitted appropriately but without clear clinical reasoning documented, which would have negatively affected our assessment of their care quality.

Although we present prospective data and our modelling adjusted for many important confounding variables, observational data has potential for residual confounding and therefore there should be caution in making causal inferences.

## Conclusion

Our nationwide analysis of the long-term outcomes of over 420 000 patients presenting with NSTEMI reveals disparities in management and mortality outcomes between those with and without a previous stroke. Individuals with a previous stroke are a higher risk cohort of patients. They are less likely to receive invasive angiography, guideline directed medical therapy, and their overall quality of inpatient care is lower. Individuals with a previous stroke had an elevated adjusted risk of mortality up to 10 years of follow-up. We show that individuals which receive excellent inpatient care had improved long-term mortality and there is potential to save over 2700 lives if these individuals received excellent care.

## Lead author biography


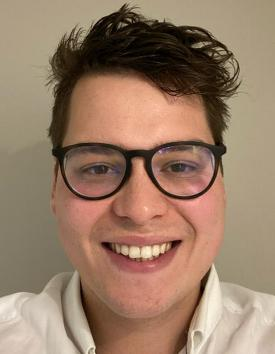


Dr Andrew Cole is a Cardiology Registrar at University Hospital North Midlands. He currently holds an NIHR Academic Clinical Fellowship and is a member of the Keele University Cardiovascular Research Group.

## Supplementary Material

oeaf143_Supplementary_Data

## Data Availability

The data underlying this article were provided by the National Institute for Cardiovascular Outcomes Research (NICOR). Data will be shared on request to the corresponding author with the permission of NICOR.
