# Supplementary material for: Effect of previous stroke on quality of inpatient care and long-term mortality risk of non-ST-segment myocardial infarction
Source: Eur Heart J Open. 2025 Oct 24;5(6):oeaf143. doi: 10.1093/ehjopen/oeaf143 (PMC12637201; doi:10.1093/ehjopen/oeaf143)
Supplement: Supplementary file 1 [file oeaf143_supplementary_data.docx]

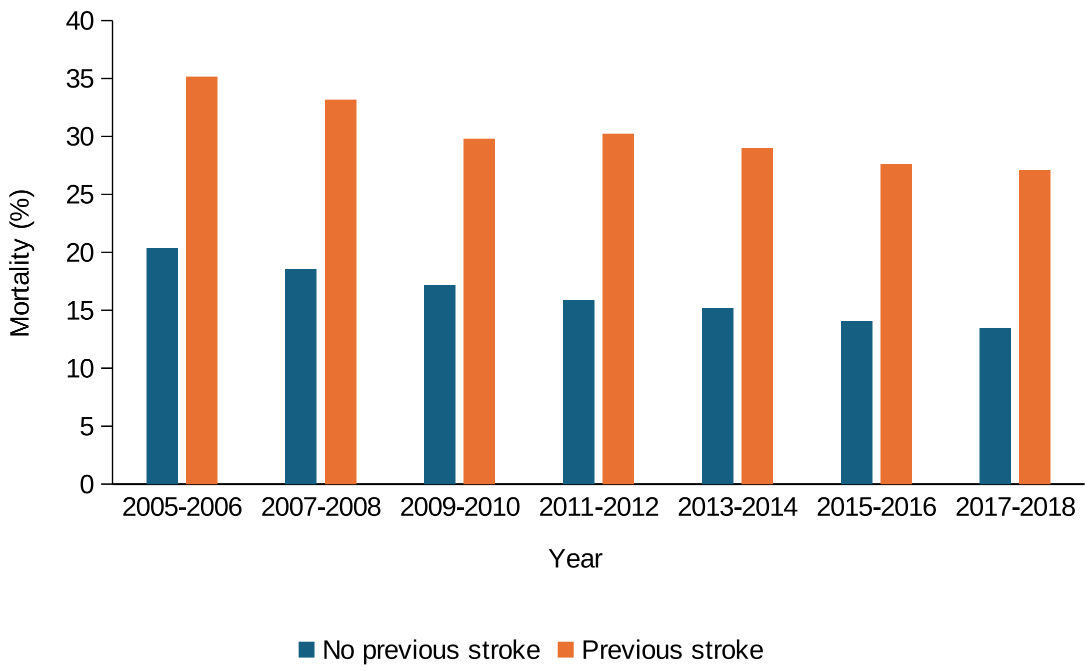

40
35
30
25
20
15
10
5
0
Mortality (%)
2005-2006
2007-2008
2009-2010
2011-2012
2013-2014
2015-2016
2017-2018
Year
No previous stroke
Previous stroke
